# Socrates’ Voluntary Death — An Essential Voice Against the Pathologisation of Suicide

**DOI:** 10.5964/ejop.19085

**Published:** 2025-11-28

**Authors:** Amadeusz Citlak

**Affiliations:** 1 Institute of Psychology, Polish Academy of Sciences, Warsaw, Poland; University of Johannesburg, Johannesburg, South Africa

**Keywords:** medicalisation and pathologisation of suicide, biography and suicide, psychobiography of Socrates, social status, structural violence, critical suicide research

## Abstract

**Objectives:**

The article focuses on the death of the Greek philosopher Socrates from the 5^th^ century BCE as a significant inspiration for contemporary discourse on suicide (an essential voice against the pathologisation of suicide). It aims to highlight how an individual and their socio-cultural environment interact when dealing with the problem of death.

**Methods:**

The psychobiographical approach used a single-case analysis as the starting point for theoretical discussion on suicide. The essential feature of the presented study is an interpretive-descriptive approach.

**Results:**

An analysis reveals Socrates’ motivations in the context of ancient Greek culture, particularly regarding the concept of honour-shame, which played an important role in the organisation of social and mental life in the Mediterranean world. However, the honour-shame dimension is not limited to ancient Greek culture; it is closely related to the universal concept of ‘social status’. Honour-shame and social status can identify and explain the motivational processes behind the suicidal decision.

**Conclusions:**

The example of Socrates (who chooses death to avoid shame and preserve his honour) allows us to receive an alternative perspective on suicide, especially concerning the right to suicide and the problem of its medicalisation and pathologisation. In this light, we should also ask for an alternative design of preventive programs and legal assessments of suicide.

## The Work and Death of Socrates

Socrates (469–399 BCE) is one of the most famous individuals of the ancient world, a renowned Greek philosopher, and a unique, vibrant personality. His motto, “I know that I know nothing”, his maieutic method of the extracting truth hidden deep within the human soul (an inspiration for contemporary therapy; Brickhouse & Smith, 2010; Pietikainen & Ihanus, 2003), and his critical attitude towards authority earned him popularity and a lasting place in history. Socrates’ life coincided with the heyday of Athens; it was a time of significant transformation, a restructuring of power relations, including the weakening of the political dominance of the Areopagus (and thus the aristocracy), which was largely transferred to the assembly of citizens — ekklēsía, represented by free Athenians. Social status was determined not so much by origin as by knowledge, social skills, and the ability to convince others of one’s views. Rhetoric played a crucial role in political and public life as one of the most effective tools for gaining influence. This new cultural model simultaneously challenged the existing system of values based on religion and tradition (Brickhouse & Smith, 2004; Ehrenberg, 2010; Johnson, 2011).

Socrates’s philosophical and educational work in such an environment automatically placed him high in the social hierarchy as an unquestionable authority, enhanced with extraordinary intellectual abilities and knowledge. His activity also had an ethical dimension, partly because he equated knowledge (episteme) with virtue (arete). Socrates was a staunch opponent of the ethical relativism typical of the Sophists of his time. However, he did not teach or enlighten others; instead, he helped to rid them of erroneous beliefs and discover the truth hidden within their souls. The pursuit of episteme is the highest human goal, regardless of the consequences (including social rejection or even death; Johnson, 2011; Moore, 2015; Smith, 2021).

Socrates’s uncompromising stance in the pursuit of truth and virtue, which he valued more than tradition or even religious beliefs, earned him numerous enemies. Socrates often overtly pointed out the flaws of democracy and state institutions, which functioned based on the voice of the majority and were susceptible to manipulation and public pressure. As he argued, “For it is not quantity but quality that should decide about the state” (*Apology*). Growing antagonism developed into resentment and conflict, culminating in the Athenian philosopher being accused of atheism and immorality, including corrupting the youth. Both accusations were ridiculous as Socrates was not an atheist, and the alleged demoralisation contradicted the very essence of his philosophical work — his ethical intellectualism (Nails, 2018).

In the first vote, the Athenian court found Socrates guilty by 281 voting against and 220 in favour of acquittal. The accused now had the opportunity to present a defence. Socrates could then have proposed an alternative punishment, such as exile or a fine (these would likely have been accepted by the court, which sought to eliminate the threat to Athens rather than to kill him). Unfortunately, Socrates just criticised the court’s decision and filed a lawsuit against it. He felt no guilt and regarded his philosophical work as a divine vocation. He therefore proposed as the “punishment” either lifelong support from the state, a privilege reserved for only the most distinguished heroes, or a fine of one *mina*, a blatant disregard for the court’s authority and its verdict. Consequently, the second vote condemned Socrates, with 361 favouring the death penalty and 140 voting for his acquittal. Thirty days passed between the trial and his execution. During this time, his students prepared an escape from prison, encouraging their master to save his own life. However, Socrates refused to escape, drank poison, and died. His death was therefore voluntary and, in a sense, suicidal (Kronska, 2001; see also Duff, 1983; Warren, 2001).

## The Question of Motivation

First and foremost, it must be emphasised that Socrates influenced the course of the trial in such a way that it could have ended in nothing but a death sentence, stemming from his conviction that flight and exile would condemn him to a life of shame, failure, and a denial of his divine mission as a philosopher-teacher (Brickhouse & Smith, 2004)^1^1In his defense (Plato's dialogue — *Apology*), Socrates also lists other reasons for his decision not to escape, such as his advanced age and the lack of means; however, these seem of secondary importance (see Brickhouse & Smith, 2004; Fagan & Russon, 2009).. The court’s guilty verdict branded him a criminal whose future work was questionable and rendered it meaningless. Socrates refused to live with the stigma of being a fraud, an atheist, and a demoralised man, especially since the trial was designed to strip him of social respect and expel him from Athens. Despite differences among historical sources that have survived to this day (Nails, 2018), the essence of the entire case was clearly Socrates’ dignity and credibility. In fact, the legal system and public authorities gave him the choice between shame and death, allowing him to preserve his honour and avoid disgrace.

Besides the above objective reasons (facts), at least two additional subjective reasons also significantly influenced Socrates’s choice. First, his personality (traits, manner of behaviour), visible, for example, in Plato’s *Dialogues*, indicates that he was a man with a high sense of personal dignity and authority. He was a proud man, “holding his head high”. The same image of Socrates is also presented in the first psychobiography of this philosopher, dating back to 1909 (and also the first psychobiography in the world)^2^2The first psychobiography is considered to be Sigmund Freud’s work on Leonardo da Vinci, published in 1910 (see Freud, 2019). In contrast, the psychobiography of Socrates was written in stages, with its first and crucial presentation appearing as early as 1909., written within the Polish school of psychology, the Lvov-Warsaw School. This school, which derives from the psychology-philosophy of Franz Brentano (Brożek et al., 2017; Woleński, 2019), alongside the Würzburg, Gestalt, and phenomenological schools, formed an important, albeit forgotten, part of the psychological milieu of the time in Eastern Europe (Citlak, 2023b). A detailed presentation of this psychobiography is available in the literature (Citlak, 2016, 2021); there is no need to repeat it here. It is consistent with what is said above and its creator, Władysław Witwicki^3^3Władysław Witwicki (1878–1948) is one of the most important figures in the history of Polish psychology, a professor of psychology at the University of Warsaw (1919–1948), author of the first Polish, two-volume psychology textbook (1926 and 1927), author of the theory of striving for a sense of power, similar to Alfred Adler’s theory, also the author of two psychobiographies: Socrates and Jesus of Nazareth (Citlak, 2021, 2025)., who analysed Socrates’s personality in the light of the social world of ancient Greece, attributes ambition and the pursuit of a sense of power/strength as the primary motivating force. In this analysis, Socrates was a fully healthy, autonomous subject who was also guided by ethical values. However, the chronologically second psychological analysis of Socrates’s personality sees him as exhibiting various disorders and dysfunctions (hallucinations, masochism, etc.; Karpas, 1915). Ambition in Socrates, as in Plato and Aristotle, had a positive meaning; it was a noble feeling that pushed a person upward, towards personal development, achievement, and competence. Ambition also fostered competition and helped individuals climb the social or political hierarchy. This motivation played a dominant role in the life of the Athenian philosopher, preventing him from cowardly escaping prison or accepting the stigma of shame. Faced with the circumstances arranged by the Athenian court, any self-respecting philosopher would have found himself in a moral and social shackle, and a personality type motivated by ambition and the pursuit of power must have found the impending humiliation and moral degeneration particularly painful.

The second subjective factor, also influencing Socrates’s behaviour, was the specificity of Greek culture, which was firmly rooted in the honour-shame dimension. Honour and shame deeply permeated not only Greek society but also Roman society (Barton, 2001; Lendon, 1997) and much of the Mediterranean basin, including the Semitic world, i.e., the Jews and Arabs (Peristiany, 1966; Peristiany & Pitt-Rivers, 1992; Pitt-Rivers, 1977). In anthropological studies of Mediterranean culture, shame had primarily social significance, referring to the relationship of an individual with a group, and to a slightly different conceptualisation of the ‘self’, namely the ‘relational self’, embedded in a network of interactions with others (Sheikh, 2014; Wong & Tsai, 2007). Shame is not identical to the individual voice of private conscience (Tangney et al., 2007). Shame means something more: disgrace, social condemnation, rejection. Severe punishments for blasphemers, such as stoning or whipping, were treated as an act of protest against the shame brought upon the community (Cross et al., 2013). Such violent public reactions resulted from shame’s primarily social, as opposed to individual, meaning (Olyan, 1996). As the opposite of honour, shame is at the other end of one dimension and is associated with losing status and recognition. Disgraced people lose respect and social position and are symbolically deprived of power. In a typical honour-shame culture, both women who were raped and men who were defeated in battle find themselves in a space of shame, and both — as numerous biographies show — may seek ways to overcome it through suicide (Canetto, 2015).

It is not only Socrates who, when pushed into the space of shame and deprived of honour, decides to commit suicide. The Carthaginian leader, Hannibal, defeated by the Romans, drank poison to avoid shame at the hands of his enemies. Queen Cleopatra let herself be fatally bitten by a snake after her husband, Antony, lost to Octavian. Mithridates Eupator, defeated by Roman armies and betrayed by his son, committed suicide (Hamilton, 2016). Acts of suicide by servants immediately after the death of their masters (as a form of honourable death) are also widely known. This also applies to suicides committed by the wives of kings, warriors, and princes (Blidstein, 2022; Lester, 1997), and was the meaning behind the Sati suicide ritual in India, during which wives died with their husbands (Hawley, 1994). There are numerous examples of suicide after losing lovers, property, reputation, etc. Historical sources show that cases of suicide due to illness or old age were quite natural, so as not to be a burden to the community or the family. In the “Age of Hellenism and the Roman Empire, a death in luxury or without suffering could be styled euthanasia. The doctor had neither a place in those acts of dying nor in cases of natural death (...) Sometimes, a doctor was called in to assist in voluntary death, a role that was not forbidden by the Hippocratic oath” (Van Hooff, 2004, p. 975). In the Semitic culture, on the other hand, a person who died in peace, after a long life and in connection with loved ones, died a so-called good death. A bad death was when one died prematurely, without burial and in violence or shame (Spronk, 2004).

Therefore, it seems that Socrates’s voluntary death was in protest of the oppressive socio-legal system, which condemned him to a life of shame and disgrace. His attitude was a dramatic, yet effective, form of defence. Shame was all the more painful and unbearable because Socrates enjoyed social and ethical recognition and was most likely a very ambitious man. However, such a case allows valuable conclusions for the contemporary conceptualisation of suicide, especially in the context of the pathologisation of suicide, directing our attention to the free and fully conscious, autonomous subject.

## The Problem of Motivation in a Biographical Perspective

Social history and literature provide many individual cases with a similar motivational context. There is an undoubted connection between suicide and mental disorders as studied in psychiatry and psychology. Nevertheless, this is only a fragment of possible explanations. Biographical studies provide important information on figures close to death or who took their own lives (Mayer et al., 2021; Misra & Srivastava, 2021; Preti & Miotto, 1999). They highlight the importance of non-medical factors, such as loss of control over life, lack of meaning in life, trauma, guilt and loss of credibility and diversity in non-medical explanations. This was shown, for example, by the research of D. Lester and J. Ill, who analysed 72 biographies of suicides in the light of the interpersonal theory of suicide by T. Joiner (Joiner, 2005), one of the most popular theories of suicide, supported by the evolutionary-biological paradigm. Joiner abstracts from the medicalisation of suicide and identifies burdensomeness (along with thwarted belonging) as a fundamental factor motivating suicides. The author refers to, among other factors, evolutionary processes and similarities between suicidal behaviour in humans and particular species of animals that withdraw from the herd when they are, for example, infected/sick, so as not to burden the other members of the group (cf. Chiurliza et al., 2018). However, Lester and Ill found that “perceived burdensomeness as a general belief that one is a burden and is characterized by a sense that others would be better off without them” (p. 1801) occurred in only 15% of study participants. An even lower result (10%) was observed by J. Gunn et al. when examining 261 suicides from Australia (Gunn et al., 2012). These results force us to consider the motivation for suicide beyond pathology and to also revise the power of explanations that accept the evolutionary paradigm as the dominant conceptual framework.

The suicides discussed above indicate the dominance of slightly different processes, especially those related to the perception of one’s actions and the assigning of meaning to them. Other biographical studies have shown that artists commit suicide less often than writers and poets (Preti & Miotto, 1999), which suggests that the sphere of artistic experience and expression may constitute a better buffer than intellectual reflection (Smit et al., 2025). Among artists (musicians, painters, sculptors), the lowest suicide rate was observed among musicians (Preti et al., 2001). In the case of most celebrities, key factors are strains in aspirations, deprivation and values (Zhang et al., 2013). The above results seem to correspond to the main claim of the psychache theory of suicide (Shneidman, 1993, 1998), in light of which the primary motivator of suicide is psychological pain resulting from the deprivation of basic human needs. Pain means “mental suffering, mental torment ... hurt, anguish, soreness, aching, misery — in the mind. *It is the pain of excessively felt shame, or guilt, or humiliation* [italics added – A.C.] or loneliness, or loss, or sadness, or fear of growing old or of dying badly, or the like” (Howard et al., 2022, p. 263).

Regardless of the possible identification of behavioural patterns or accepted theories of suicide, one of the most important motivating factors is the subjectively stated lack of possibility of a dignified, valuable life. This becomes an essential source of psychological pain, motivating people to end their lives, regardless of whether we are talking about inhabitants of the Eastern or Western world (Snowdon, 2018). And this problem is becoming increasingly felt today due to technological and medical achievements, which, while allowing us to improve the quality of life, force us to question its value and necessity in extreme circumstances, such as suffering, pain, loneliness, lack of a sense of meaningful life, etc. It is precisely the connection between suicide and voluntary death with the concept of dignity that is a kind of constant, as evidenced by literature, history, mythology, religion and anthropology (Lu, 2017). One of the more misleading conclusions in the assessment of suicide and its pathologisation is the reliance on correlations between it and the concept of dysfunction (e.g., depression, anxiety). By its very nature, this correlation only suggests but does not determine causal relationships. It is evident that the lack of a sense of meaningful life and dignity is key to mood disorders (anxiety, depression), and they are the most significant challenge for both people planning suicide and survivors (Aviad-Wilchek & Ne’eman-Haviv, 2018; Zhang et al., 2023).

It has also been repeatedly emphasised in the literature that a particularly important factor in suicidal acts is the experience of aggression (usually aggression experienced by young people and traumas related to aggression in older people), which may contribute to problems with depression or anxiety (McCloskey & Ammerman, 2018). However, the issue of suicide gains new meaning when we consider that aggression can take various forms, from incidental behavioural and psychological acts to systemic oppression and institutional violence. Suicide as a result of institutional, systemic aggression becomes an act of protest, an attempt to regain control, strength, and above all, dignity. It becomes a rallying cry for a dignified life and death (Citlak, 2023a, 2024). This applies to both cases set in individual contexts (personal drama) and group, social or political contexts (Abdeen et al., 2018; Nugent & Lee, 2025), with self-immolation in protest against oppression, rape, violence, suicidal acts of samurai as the only way to avoid shame, kamikaze as a form of struggle to maintain the honour of the group, and suicides among women raped during war being notable examples (Lamb, 2020). Even terrorist suicide attacks can be (and unfortunately are) inscribed in the perspective of the struggle to maintain honour and defence against shame (Beller & Kröger, 2021; Greenland et al., 2020).

## Between Honour-Shame and Social Status

The concepts of honour and shame play a key role — often underestimated in contemporary WEIRD cultures — in the motivation for suicidal acts and the desire for a good death (Cameron et al., 2020). Historical sources, philosophical and literary texts clearly show that loss of dignity usually became one of the leading causes of suicide, as is also the case with the desire to avoid shame. More recently, anthropological studies of Mediterranean culture conducted in the 1960s and 1970s showed the importance of the honour-shame dimension first among the inhabitants of the Iberian Peninsula and then in Italy, Greece, Egypt, Turkey and North African countries (Peristiany, 1966; Pitt-Rivers, 1977). This dimension regulates the lives of the vast majority of the inhabitants of the Mediterranean basin (Peristiany & Pitt-Rivers, 1992). It concerns Jewish and Muslim cultures, as well as the countries of Southern Europe. Nowadays, the great significance of honour-shame is found in the culture of the Middle East and many countries in Africa, Asia and North and South America (Miedema et al., 2020). This fact is not a weakness of the proposed model but a confirmation that honour-shame is a constitutive feature of societies with a relatively high intensity of collectivism and collectivist ethics (Guerra et al., 2013). However, historical and cultural changes have systematically led to an increase in the distance between the individual and the community, favouring the process of individualisation of the subject, and as a further consequence, ethical changes and the development of new social relations (Tomasello, 2009). This process began in ancient Greece, but the individualisation of the subject, as we know it from Western culture, accelerated after the collapse of the dominance of the church in Europe, the influence of the Enlightenment, and then, as a result of industrial development and democracy (Ferngren, 1989). Individualisation does not negate the value of the honour-shame dimension. It still plays a vital role in social relations, although not as dominant as before. In other words, honour-shame cannot be treated as belonging to the past or exclusively to collectivist countries. As an immanent component of interpersonal relations, it has moved over time from the central field of key values or been replaced by concepts/values with a similar function (Guerra et al., 2013; Helkama et al., 2013). From a historical, evolutionary perspective, shame is still present and plays a similar role, although it entails a more personal sense of shame in the WEIRD (individualistic) culture, as a result of negative evaluation in one’s own eyes and less in someone else’s (Lester, 2008). Similarly, honour meant not only dignity and social recognition but also provided a higher position in the community hierarchy, and thus the possibility of exerting influence and a sense of power. In the WEIRD culture, however, the honour experience’s connection with social evaluation has weakened, leaving a stronger connection with a personal sense of pride, ambition and self-esteem. I draw attention to this because the honour-shame dimension seems to be constantly present as a fairly broad category, adopting a slightly different expression or linguistic grid (Landers et al., 2024; Sznycer et al., 2018).

Perhaps we should even speak of a single universal motivational factor instead of a bipolar dimension. For example, instead of honour-shame, we could refer to the concept of psychological strength-power, which emerged in the history of psychological research at the beginning of the 20^th^ century. It would be about a sense of strength, agency, and status, the relatively high intensity of which is expressed by honour and low by shame. As an example of this, A. Adler’s *Individualpsychologie* offered a coherent conceptual framework, with *Machtegefühl* and *Machstreben* as the essential human motivational factor (Adler, 2000, 2011; similarly, the first European power-strength motivation theory — Citlak, 2025). At the social level, some suggestions are provided by the social dominance orientation theory of J. Sidanius and F. Pratto (Sidanius et al., 2004), in the light of which, the orientation towards dominance is a universal feature (Pratto et al., 1994). I believe the extent to which such or similar strivings may correlate with positive (compassion, altruism) or negative (discrimination, violence) behaviour is not so important. Particular theories emphasise different aspects of possible correlations. The above and similar theories must refer to a similar factor regulating social life. Comparing the importance of honour-shame in the ancient and contemporary worlds, C. Patterson argues that honour can be treated as an expression of a universal variable, i.e. social status (Patterson, 2019). In both cases — honour and social status — we are motivated by social comparisons. In fact, ancient and modern honour would mean nothing more than a form of striving to maintain or increase status (van Osch et al., 2020).

In the context of suicide and good death, this means that the problem of evaluation and self-image, both in the eyes of the environment and oneself, was and remains a fundamental issue. Moral valuation (evil), religion (sin) or pathologisation (disease, disorder) should be seen as secondary phenomena imposed externally in an attempt to universalise the subjective experience originally embedded in sociocultural canons and local contexts. Even if we take into account the indigenously diverse, local conceptualisations of suicide and good death, it seems that they are connected by similar motivational factors embedded in the experience of shame, guilt, loss of dignity or the status of a subject who no longer fits in the public space or the system of personal beliefs and values. Although this applies primarily to those suicides that do not result from diseases or disorders, in fact, many of the latter also fit this perspective. Noteworthy in this respect are the experiences of V. Frankl working with people who had experienced trauma and lost the meaning of life; he helped them abandon suicidal intention by finding a sense of meaning and personal dignity (Frankl, 1959; Kleiman & Beaver, 2013).

## Beyond Medicalisation and Pathologisation of Suicide

Medicine has treated suicide as a manifestation of mental dysfunction or disorder since at least the 17^th^ century. Pathologisation replaced former categories of evil, sin or possession. The secularisation of suicide assessment (Weaver & Wright, 2009) was often expressed in the Latin *non compos mentis* (not of sound mind). In the 19^th^ century, suicide was usually seen as an illness or a form of madness (Houston, 2009). Today, we estimate that at least 50% of suicides are committed by people diagnosed with personality disorders, depression, deep anxiety or schizophrenia (Marsh, 2010). Medicine indicates a neurobiological basis for suicides: “Serotonergic, noradrenergic, glutamatergic and GABAergic systems, as well as the HPA axis, are the main neural networks involved in the pathophysiology of suicide. Disorders of opioid and endocannabinoid systems can also be found in suicide victims. Pathogenesis of suicidal behaviour also contains abnormalities of cell signalling and pathology of glial cells” (Jaeschke et al., 2011, p. 573). From a medical perspective, however, it remains difficult to answer the question of whether the specific constellation of neurobiological processes in suicides should be treated as a separate entity of disorders or rather as their integral part (Hannon et al., 2025). Perhaps these constellations are also, to some extent, the result of the impact of situational life factors. Regardless of this, psychiatry treats suicide unequivocally as a dysfunction, as a suicidal behaviour disorder (DSM-5, 2013), and plays a decisive role in its pathologisation. In many works, the authors directly state that mental disorders and psychopathology are the main conditions for suicide. Psychology, especially clinical psychology, also actively participates in the pathologisation of suicide, indicating numerous connections not only with disorders such as depression, anxiety and schizophrenia, but also with selected personality traits (Lu, 2017). This point of view is of decisive importance in the approach to people affected by the problem of suicide. If a threat to the patient's life is determined, there is an obligation to treat, including hospitalisation. As a consequence, this triggers a complex process of coercive influence, legal procedures, and often secondary medical and social stigmatisation (Rimkeviciene et al., 2019).

Münster and Broz (2015), in their anthropological analysis of suicide, point out that while “most human societies tend to treat suicides not as idiosyncratic and exceptional ways of dying but as a kind of death indicative of larger, more significant issues, such as ruptures in social, economic, and moral life” (p. 5), unfortunately, medicine and psychology do not sufficiently take this fact into account (cf. Staples & Widger 2012). These disciplines are additionally aided by statistical studies of the dependencies or correlations related to suicide, by analysing and aggregating huge amounts of quantitative data (e.g., demography, sociology, public health). Their constitutive feature is the transition from the qualitative level, related to the subject’s experience, to the level of average, general dependencies between variables (serotonin levels, gender, genetics, depression, financial crises). This separates the experience of suicide from the subjectively and socially created meaning/sense of the act itself and weaves it into identifiable statistical relations, resulting in reductionism and the support for the aforementioned pathologisation of suicide. Medicalisation thus rationalises the pathology of suicide, while quantification indicates correlations between it and health indicators. Münster and Broz call these (medicalisation and quantification) ‘expert knowledge regimes’ and rightly point out how the lack of an adequate definition of personhood in Western science (i.e., WEIRD) results in the inadequacy of defining suicide, as individualism does not sufficiently take into account the social, cultural and local networking of the self (Staples, 2015). Ultimately, “suicidology (…) assumes that the persons inhabiting the suicide field exist prior to the act of suicide” (p. 16). This is classic ‘psycho-centrism’, locating problems and their solution within the psyche (Jaworski, 2014) and overemphasising the subject’s agency. Other researchers draw attention to the rich semantic background of suicide and voluntary death, which is interwoven with religious and ethical systems, local philosophy and rituals — unique systems of culturally constructed meanings (Marsh, 2010). This is evidenced by numerous examples, including the populations of India, Palestine, Siberia, indigenous groups of Australia, Africa and North America (Brooke et al., 2025; Lester, 1997; Reyes-Foster, 2015; Sharp & Linos, 2015). The concept of disease or disorder is only a marginal part of the phenomenon in question and does not consider the extensive connections between the subject and environmental dynamics. An indigenous approach to suicide and voluntary death, i.e., studying them in a local context, taking into account the interpretative canons of a given community at a given time, forces us to go beyond narrow medicalisation to broaden the field of possible explanations, such as political rebellion, moral opposition, religious obligation, dynamics of power relations, etc. This point of view is also present in critical suicide research.

It is worth adding a few words about the religious stigma of suicide. Western ethical thought has been shaped largely by monotheistic religions, where life is a gift from God, so the human in question cannot decide when to end it. Suicide or euthanasia as a voluntary act of taking life is considered a sin, an offence against the will of God; in Judaism and Christianity, it is an offence against the commandment “Thou shalt not kill”. The Jewish Talmud allows for refraining from medical practices in a situation of imminent death and artificially sustaining life. Suicide rates among followers of Judaism are one of the lowest in the world (Gearing & Alonzo, 2018; Saiz et al., 2021; Wilchek-Aviad & Malka, 2016). Augustine played an essential role in the Christian view of suicide, as he considered it a sin worse than murder, because the criminal no longer has a chance to repent (*De Civitate Dei*). The prohibition and condemnation of suicide also occur in Islam (“Do not kill one another”, “Do not kill” — Surah IV) as a crime equal to murder, and which cannot be forgiven. According to the Quran, life is a gift from God, and only he decides to end it. Muslim countries have the lowest suicide rates in the world (Arafat et al., 2022; Lew et al., 2022; Shoib et al., 2022). However, I would like to emphasise an important yet commonly marginalised fact that monotheistic religions additionally created an evident space for the acceptance of suicide under extreme conditions. This primarily applies to the early period of Judaism and Christianity. In the context of suicide and dignified death, it is particularly important that the Semitic culture, where monotheistic religions were born, was dominated by the dimension of honour vs. shame (Citlak, 2023a). The prohibition or acceptance of suicide in monotheistic religions has always been part of a broader religious and ethical context. Moral norms had a strong historical and cultural background; for example, the prohibition of killing did not include enemies who destroyed the sacredness of Israel or Muslims, because the sacred was a superior value to the specific commandments (Loewenthal et al., 2003).

The dimension of honour vs. shame had a decisive impact on the moral assessment of suicide and dignified death in monotheistic religions. Both phenomena were closely connected. All suicides described in the Old Testament are related to the desire to maintain honour or avoid shame (Koch, 2005). King Saul asks for a dignified death from his servant to avoid humiliation and shame at the hands of the infidels; when the servant refuses, Saul kills himself. The biblical Samson commits suicide to avoid shame at the hands of the pagans. None of them were condemned in the Bible (Shemesh, 2009). Suicide committed to avoid shame and preserve honour was simply accepted, and it has such a moral status in the holy books of Judaism and Christianity. Moreover, people already dishonoured due to unworthy acts were also not condemned when they committed suicide, which was a natural consequence of their shame, as if expected by the community (e.g., King Zimri, Ahithophel). Judas’s suicide is similarly presented in the New Testament as a ‘natural’ decision resulting from a shameful betrayal. The later rabbinic tradition accepts (or at least does not condemn) voluntary death and suicide in exceptional situations, for example, if it were to protect the believer from denying God or from blasphemous acts during persecution or torture (Witztum & Stein, 2012). During the persecution of Christians in the 2^nd^–4^th^ centuries, many people, including bishops, chose voluntary death and public execution at the hands of the executioners rather than a shameful escape (Moss, 2012). A similar meaning in radical Islam is found when suicide attempts to preserve the honour of the family or community. Then, such an act is understood as “a form of resistance” as an opposition to “humiliation and defeat” (Abdel-Khalek, 2004, p. 109). Unfortunately, such an interpretation is cynically exploited by terrorist groups (Slavicek, 2008). In other words, suicide and dignified death in circumstances defined by honour or shame had a place in these religions as part of a wider system of social interactions. Despite the prohibition “Thou shalt not kill,” not all suicides were subject to condemnation; they could be accepted and sometimes even expected.

## Critical Suicide Research

The above reflections allow us to look at suicide and the dignified death of Socrates (and many other people today) in a much broader perspective than traditional suicidology offers. In many respects, similar proposals for a wider view are presented by the critical suicide research: “Critical Suicide Studies developed across the 2010s as a means to theorize and intervene in suicide from perspectives alternative to the ‘mainstream’ of suicidology (...) ‘mainstream’ or ‘traditional’ suicidology can be characterized as originating in two key ways: a North American ego-psychological framework that posed suicide as an individual act resulting from poor mental health, and a European model that approached suicide as social, the trends and causes of which are primarily apprehended through statistics” (Chandler et al., 2022, p. 3). This goes beyond the limitations of the biomedical and individualistic framing of suicide. Representatives of this approach draw attention to the basic problem of the often unjustified shift of attention from the context to the autonomous intentional subject, with preventive programs accordingly underestimating external or structural factors as they focus mainly on repairing the individual psyche. In order to go beyond these limitations, they propose to revise such beliefs as:

- Inadequate understanding of the self as an autonomous, individualistic entity.
- Psychocentric understanding of distress (cf. Marsh, 2020).
- Scientific knowledge production, which is never independent and objective but is always part of cultural, political, discursive assumptions — this applies to both qualitative and quantitative, experimental research (Bantjes & Swartz, 2017).
- The specificity of prevention as dependent on scientific knowledge (White, 2017).

The lack of such awareness means that “social problems are individualised and privatised and [social, political, cultural — A.C.] inequalities are converted into forms of pathology (...). Meanwhile, structural forms of violence, racism, or heteronormativity are obscured from view” (White, 2017, pp. 475–476). The author also cites the telling example of suicide among the indigenous population of Canada in the context of inadequate traditional prevention programs that do not take into account political oppression, cultural genocide, etc. (Kral, 2012)^4^4Critical suicide studies, therefore, take into account the broad context of suicide, such as “social exclusion, colonial violence, racism, patriarchy, the role of consumerist and transactional health practices and other injustices.” (Chandler et al., 2022, p. 3).. A more adequate approach to suicide (and dignified death) must consider collective ethics and political responsibility, in the light of which suicide is also a social act. The ethical aspect is formulated in a new way, referring to a problem with a particular repressed ethical dimension in the traditional discourse: “It means raising questions about whether some suicides might be justifiable in a societal context” (p. 473). This radically changes the way of thinking and talking about suicide, “no longer understood as a giving up on life or the opposite of life (and thus something to be forbidden)”, but in some circumstances “as particular forms of life or life-activating practices that provoked vital critique, freedom, rebellion, solidarity, and transformation” (p. 478).

Essential conclusions are also formulated by J. Ansloos and J. White (Ansloos & White, 2025), presenting various forms of alternative therapy for people affected by suicide (including their families) as Counter-Institutional Models of Care. By way of example, TransLife, DISCHARGD (Deserving of Inclusion, Support, Community, Hope, Authenticity, Respect, Growth, Empathy, and Determination), The Big Anxiety Research Centre, and Slice/Silence, create counter-institutional spaces in which people affected by suicide can experience suicidal distress outside of the traditional clinical scheme and ethical or legal assessment^5^5It is difficult to determine the effectiveness of these models at this stage; however, they offer an alternative form of support that is close to the essence of suicide, taking into account its social and political roots. More information, see e.g., Cesar Riani Costa & White, 2024; https://translifeline.org.. Survivors have “the chance to engage directly with their experiences and reclaim narratives that are often suppressed or medicalized in traditional settings” (Ansloos & White, 2025, p. 19). Some proposals for support “advocate for an abolitionist counter-space for psychotherapy for suicidal individuals, shifting safety planning away from police involvement (...). These initiatives reimagine suicide response as an act of solidarity rather than containment, focusing on collective care rather than coercive intervention” (p. 20)^6^6The authors define counter-institutions as an “alternative spaces operating outside mainstream systems that foster autonomy, collective care, and self-determination. Grounded in prefigurative politics, these counter-institutions enact an “ethics of listening” (…), prioritizing dignity, agency, and relationality over control and coercion” (Ansloos & White, 2025, p. 18).. This approach expresses a unique scientific and therapeutic ethos described in the Statement of Ethics by the Critical Suicide Studies Working Group (CSSNWG, 2019). It includes the principles and beliefs that structural violence creates “vulnerability to suicide”, and this particularly applies to racialised, minoritised, and indigenous people^7^7Similarly H. Hightower (2024): “Black suicides result from interactions between macrosystemic systemic forces and individual-level meaning-making processes” Author identified “six thematic characters for critically understanding suicide in Black communities: shame, hopelessness, trauma, racism, systemic problems, and fear” (p. 151).; that critical research must “respect the agency of people who choose suicide, and will not live”; furthermore, “to acknowledge the complexity of suicide prevention and does not take it for granted as an unqualified good.^8^8https://criticalsuicidestudies.com/what-is-the-critical-suicide-studies-network (retrieved 10.06.2025).” Suicide prevention should be dependent on the local, indigenous context.

It is not necessary to explain that the perspective of critical suicide research is a complementary proposal to understand Socrates’s death. One of the main postulates of this trend is the analysis of suicide in the context of systemic oppression, which, by depriving an individual of a sense of dignity, pushes them into the space of shame. Preventive programs are supposed to restore dignity to people or groups affected by the problem of suicide, not only on the individual level but also on the social and cultural, political level.

## Conclusions

The story of Socrates, like many biographies from the ancient world, transcends the concept of pathology due to the close connection between suicide and the sense of honour-shame. In many cases, it is only then that they become understandable and escape negative moral judgment. Honour and shame, closely related to the concept of social status, continue to play a significant role in social life and should not be overlooked in the analysis of such critical decisions as the desire to end of life, even in the 21^st^ century. The example of Socrates also clearly opposes the uncritical and not always necessary medicalisation of suicide in psychiatry and psychology (Botes & van Niekerk, 2025). Medicine typically categorises it as a disorder requiring treatment (also obligatory) and stigmatises the suffering people. Medical discourse (alongside religious discourse) not only overemphasises the agentic nature of the subject and underestimates the local indigenous context, but at the same time, is the most important source of the pathologisation of suicidal acts, directly influencing the design of preventive programs and legal assessments.

An analysis of the biographies of Socrates and many other individuals affected by suicide — aside from cases of disorders and illnesses — demonstrates that, despite the passage of time, one of the fundamental problems associated with suicide remains the loss of dignified life, in addition to positive self-esteem and social esteem. I have attempted to demonstrate that these can be treated as a kind of expression of the traditional honour-shame dimension, as well as the more geographically and historically universal concept of status. Such an interpretation is, of course, a theoretical proposition and would require more detailed conceptual analysis or empirical verification. However, biographical examples, the need to transcend the medicalisation and pathologisation of suicide, and the rich empirical evidence in suicidology, allow us to adopt a broader perspective and seem to support this proposition.

The presented analysis leads to the conclusion that suicide can be understood as the result of oppressive power dynamics and circumstances that deprive individuals of their sense of dignity and status, forcing them into a space of shame. The same oppressive processes we see in the case of repressed ethnic, social or sexual minorities appear to play a key role in suicides without mental disorders (the very disorders may be only a consequence of repression). Our perspective radically changes, firstly, the moral assessment of suicide, escaping the category of evil, sin, or crime. Secondly, it also requires a new design of preventive action, focused on the broadly understood empowerment of those affected by suicide, without penalisation, stigmatisation, or coercion, which are often merely an expression of oppressive (pathogenic) power dynamics.

## Data Availability

The analysed historical/cultural sources are available in the public domain.
